# The Importance of Accurate Diagnosis of ME/CFS in Children and Adolescents: A Commentary

**DOI:** 10.3389/fped.2018.00435

**Published:** 2019-01-21

**Authors:** Keith James Geraghty, Charles Adeniji

**Affiliations:** Division of Population Health, Health Services Research and Primary Care, Centre for Primary Care, University of Manchester, Manchester, United Kingdom

**Keywords:** chronic fatigue syndrome (CFS), myalgic encephalomyelitis, diagnosis, prevalence, pediatric case

## Abstract

Myalgic encephalomyelitis/chronic fatigue syndrome (ME/CFS) is a chronic illness that causes a range of debilitating symptoms. While most research has focused on adults, the illness also presents in children and adolescents. Many physicians find it difficult to diagnose the illness. In this commentary paper, we discuss a range of salient themes that have emerged from our ongoing research into the prevalence of ME/CFS in children and adolescents. We discuss reasons why pediatric prevalence estimates vary widely in the literature, from almost 0% to as high as 3%. We argue that there is considerable misdiagnosis of pediatric cases and over-inflation of estimates of pediatric ME/CFS. Many children and teenagers with general fatigue and other medical complaints may meet loose diagnostic criteria for ME/CFS. We make recommendations for improving epidemiological research and identifying pediatric ME/CFS in clinical practice.

## Introduction

Children and adolescents with suspected myalgic encephalomyelitis/chronic fatigue syndrome (ME/CFS) regularly present with persistent fatigue, sleep disturbance, and an array of other symptoms, such as headaches and cognitive difficulties (1). ME/CFS is noted for being a major cause of long-term school absence and has profound negative ramifications for social development, educational achievement, and future employment (2, 3). The illness is associated with co-morbid anxiety and depression (4). It is known that children with chronic health problems exhibit higher rates of distress, anxiety, and depression (5). Taking these factors together, it is vital that young patients with this illness are correctly identified, so that they might receive a speedy diagnosis and appropriate medical care and social support.

Epidemiological studies report a wide range of prevalence estimates of ME/CFS in this age group. Some estimates are as low as 0.1% (6), while others suggest rates of 2.6% (7); and rates for CFS-like illness go as high as 4.4% (8). Girls are at greater risk of developing ME/CFS, particularly post-puberty (9). This wide spread in prevalence estimates appears to result from researchers using different diagnostic criteria to classify cases and applying different methods to sample and identify cases, such as postal or telephone questionnaires, community-based surveys, and clinical interviews. Given the general lack of consistency in methodologies applied, inconsistency in prevalence estimates is not surprising. However, such inconsistency suggests a problem with the methods used to identify young ME/CFS sufferers. It is clear, with estimates as low as 0.1% and as high as 3–4%, many young patients are being misdiagnosed, either under or over. Misdiagnosis in this vulnerable group has profound implications, since a false positive diagnosis may lead to inappropriate labeling of a child with ME/CFS and improper intervention with treatment (10), while under-diagnosis might mean a child or teen not receiving the care they require. If researchers are unable to reliably identify pediatric cases of ME/CFS, how confident can we be that clinicians are able to diagnose cases at the clinic level? We know doctors often find it difficult to diagnose ME/CFS and adult sufferers commonly wait an average of 5 years for a diagnosis (11).

## The Root of the Problem

The International Chronic Fatigue Syndrome Study Group Criteria (12) is one of the most cited in the literature. The Fukuda Criteria requires severe and disabling new-onset fatigue lasting at least 6 months, accompanied by four or more of eight symptoms: impaired memory or concentration, sore throat, tender cervical or axillary lymph nodes, muscle pain, multiple joint pain, headaches, unrefreshing sleep, and post-exertional malaise (PEM). However, in the UK an alternative case definition, known as the Oxford Criteria (13), is promoted, that is much broader, given it only requires a single symptom—severe and disabling fatigue of definite onset that is present for at least 6 months and affects physical and mental function. Other symptoms often found in ME/CFS patients, headaches, sleep problems, orthostatic intolerance and so on, may be present, but are not required to be diagnosed with ME/CFS using Oxford Criteria.

In 2015, a report by the US Institutes of Health found the Oxford Criteria too broad to be of value in investigations of ME/CFS (14). The report stated that use of this case definition could impair progress and cause harm by conflating fatigue as a complaint with the illness ME/CFS. The Fukuda case definition has also been criticized; while it requires the presence of other symptoms to render a diagnosis, it does not specifically mandate that patients experience post-exterional malaise (PEM), which is considered a cardinal symptom of the illness. There are newer case criteria for ME/CFS, such as the Canadian Consensus Criteria and the International Consensus Criteria or the U.S. Institute of Medicine (now known as the National Academy of Medicine) Systemic Exertional Intolerance Disease formulation, that require the presence of PEM, however there continues to be a lack of consensus on which diagnostic criteria should be used (15). Researchers studying children or teens with the illness arbitrarily select a criterion to identify cases.

Most research in ME/CFS has focused on adults with the illness. In many adult studies, broad case definitions that require little more than fatigue as the presenting complaint, have been used to recruit patients into clinical trials of treatment interventions; commonly psychological and behavioral treatments, such as cognitive behavioral therapy (CBT) and graded exercise therapy (GET). For example, the largest clinical trial of psycho-behavioral treatments in adults, the UK PACE trial, tested CBT and GET against standard medical care and a pacing therapy (16). The PACE trial reported CBT and GET to be moderately effective compared with pacing treatment or standard medical care. However, recent commentaries have questioned whether PACE recruited true-positive ME/CFS cases (17, 18)—-the Oxford Criteria was employed to select participants. Recent reanalyses of data from the PACE trial suggests treatment benefits were grossly over-stated (19).

Another major problem in this field of research is the ubiquity of “fatigue” or “chronic fatigue” as a medical complaint and its conflation with “chronic fatigue syndrome.” Pediatric studies of ME/CFS that apply broad diagnostic criteria may recruit cohorts with generalized fatigue, rather than cohorts with the cardinal symptomology of myalgic encephalomyelitis (20), proposed by Ramsay (21). The Oxford Criteria requirement to only need ongoing fatigue as a presenting complaint means many young patients with general fatigue issues could be misclassified as having ME/CFS. Up to 20% of adult patients seen in community/primary care settings present to doctors complaining of fatigue and up to 33% of adolescents experience fatigue at least 4 days per week (22, 23). UK community doctors are encouraged to refer young patients with suspected ME/CFS to be treated within specialized CFS clinics that offer CBT and exercise therapy (24). However, for adult ME/CFS patients referred to these clinics, there is a diagnostic error rate of at least 40% (25, 26) and the majority of patients treated (90%+) still report having ME/CFS at long-term follow up (27). In one of these clinics, many patients were eventually diagnosed with other conditions to explain their fatigue; 47% being diagnosed with a chronic disease, 20% a primary sleep disorder, 15% a psychological/psychiatric illness (most commonly, depression, anxiety, and post-traumatic stress disorder), and 4% a cardiovascular disorder (26). Community doctors find it difficult to differentiate fatigue linked to undiagnosed medical or mental health complaints, from clear ME/CFS.

A series of epidemiological studies into ME/CFS prevalence in teenagers conducted at the University of Bristol used a birth cohort database called the Avon Longitudinal Study of Parents and Children (ALSPAC). This database includes information on 14,500 families from Bristol and the surrounding region, with health status monitored through self-reported questionnaires filled out by both parents and children. The ALSPAC database, in conjunction with follow-up questionnaires, has been used to assess pediatric chronic fatigue prevalence, with rates reported of 1.47% at age 13 years, 2.22% at age 16 years, and 2.99% at age 18 years (28). Here “chronic disabling fatigue” is used as a proxy measure of chronic fatigue syndrome. In one of these studies published in *Pediatrics*, 41% of parents (n2201) reported their teenager being tired or lacking energy in the last month. Clearly, fatigue is a common complaint among adolescents. Of 2,201 possible CFS cases identified, after exclusions (e.g., fatigue not causing loss of activity), 4.17% (n207) with fatigue > 3 months and 2.76% (n137) with fatigue > 6 months were classified as possible cases (29). After a “Life at 16 Questionnaire” was administered to this cohort to match 16-year olds with self-reported fatigue–this generated a CFS prevalence estimate of 1.9% (29). Across the ALSPAC studies, estimation of prevalence uses proxy measures of CFS (chronic disabling fatigue), parental reported fatigue, self-reported fatigue and or school absence; however, there is a lack of detailed clinical screening or the requirement for cardinal symptoms of ME/CFS to be present, such as post-exertional malaise. As such, the near 2% prevalence rate to emerge from the ALSPAC studies, is likely to be an over-estimation of pediatric ME/CFS.

## Not all Fatigue Is the Same

Since fatigue is a common complaint among children and adolescents and up to half of all parents perceive their children to have “a problem” with fatigue (29, 30), there is a clear need for robust clinical investigations to assess the causes of presenting fatigue in young patients–whether it is the usual fatigue many teenagers experience, or whether it is the type of fatigue that is characteristic of ME/CFS (not all fatigue is the same). Any methodological approach that conflates the symptom of fatigue with ME/CFS is likely to inflate case estimates. For example, in the 2.99% prevalence rate of chronic disabling fatigue reported at age 18 years (28), only 29% of this CDF cohort meet the US CDC/Fukuda criteria for CFS; whereas presumably most would meet UK guidelines for CFS (31). In UK pediatric prevalence studies that apply the CDC criteria, pediatric prevalence falls to 0.019–0.05% (32, 33). This is an illuminating finding.

In the Crawley et al. prevalence study of chronic disabling fatigue at age 13, only 30.7% of teens identified as possible CFS cases had presented to a doctor complaining of fatigue (34). Presumably, the other 69.3% didn't feel their fatigue was related to a medical condition like ME/CFS, that required medical attention. Even when children or teenagers (most likely with concerned parent) present to doctors complaining of fatigue, a diagnosis of ME/CFS requires a triangulation approach, using multiple strands of information to build up a clinical case profile that helps exclude other potential medical or psychological conditions (35). Where careful clinical screening is applied, with clinicians undertaking a detailed case history, laboratory tests or psychological screening, pediatric prevalence rates fall to as low as 0.1% (6) or 0.06% (36).

Depression, mental health complaints and substance abuse, are a major cause of unexplained fatigue in young ME/CFS patients (6), thus there is a clear need for pediatric patients to be carefully screened before being given a ME/CFS diagnosis (37). The difficulty for any physician will be how to differentiate co-morbid depression and anxiety from primary depression or anxiety, as the cause of presenting fatigue. Failure to robustly assess mental health as a possible cause of fatigue is likely to lead to inflated estimates of ME/CFS. In the study of CFS rates among 16 year-olds, rates of CFS fell by more than two-thirds, from 1.89 to just 0.6%, after investigators removed those with high levels of depressive symptoms from their analysis (29). This lower 0.6% figure is much closer to rates of ME/CFS reported among adults, which commonly fall between 0.1 and 0.5% (38).

## Implications for Treatment

The problem with over-estimation of pediatric ME/CFS is an epidemiological one that is likely to impact resource allocation and health planning. However, misdiagnosis at the clinic level is even more concerning–many children and teenagers may be wrongly diagnosed with ME/CFS. These young patients will most likely trust a diagnosis given by a physician and they are likely to follow recommended care, which might include being offered psycho-behavioral therapies like CBT or graded exercise (which are recommended based on clinical trials that apply the same loose diagnostic tools that generate inflated prevalence estimates). The Bristol ALSPAC research team, that report prevalence rates as high as 2%, are active in testing CBT and GET on children and teenagers with suspected ME/CFS (39, 40). There is a concern that psychological therapies may help teenagers that perhaps have undiagnosed psychological complaints or general fatigue complaints, who are inappropriately included into clinical trials. Basically, many teenagers with general chronic fatigue issues may meet UK Oxford/NICE criteria for ME/CFS. However, data on the success of these therapies is contaminated by the inclusion of significant numbers of false-positive cases. This concern might be evidenced in data from the ALSPAC studies that show that only 11% of teens identified as possible ME/CFS cases continued to have a problem with chronic disabling fatigue over two time points: 85.25% (6 months fatigue) between the ages of 13–16 years recovered and 79.80% (6 months fatigue) between the ages of 16–18 years recovered (CMRC Conference Presentation on ALSPAC recovery rates 2014). Essentially, 8 out of every 10 teens identified as possible CFS cases recovered by age 18 (or were wrongly classified as CFS).

A current large clinical trial (FITNET) of internet-based CBT and tele-support with activity management for teenagers (age 11–17 years) with ME/CFS uses broad (Oxford/NICE) criteria to select participants (40). A major justification used by the trial team is that teenagers have a 63% chance of recovery using FITNET vs. just 8% chance using standard medical care (40). This data is taken from a pediatric CBT trial of FITNET in the Netherlands (41). The Dutch trial has been criticized for over-stating benefits via *post-hoc* selection of recovery measures and for including young patients with general fatigue issues (42). Interestingly, at long-term follow up in the Dutch FITNET study (2 years+), recovery stood at 64% for CBT-GET participants, but 52.8% for usual care participants (43). Remarkably, teenagers in the standard care group (which is often nothing more than usual general practice care) improved over time, with relatively little difference between the CBT cohort and the *de facto* no-treatment control. This same phenomenon is visible in adult CBT trials, with the gap closing between the intervention and standard care in the PACE trial (44) and FINE trial (45). What we can take from this observation is that CBT or exercise therapy perform little better than no care over the longer term. There are other reasons some trials show modest benefits over the short-term, such as selection of milder cases and strong treatment promotion effects (18, 46). Taking this into consideration, in addition to noting high rates of natural recovery in children and adolescents, the case for early intervention with psycho-behavioral therapy is rather weak. Good quality primary care support should always be available. For more severe cases, treatment in specialist secondary care should be available also. This care should include symptomatic support, advice on nutrition, sleep support, pain control, infection control, allergies, and mental health issues (35). There is no evidence to support GET for severe ME/CFS cases (clinical trials do not include severe homebound sufferers). Very little is known about patients with severe ME/CFS. They are often housebound, bedbound and are rarely studied. Overall, much more research is needed around all aspects of pediatric ME/CFS.

## Conclusion

There is a clear need for robust prevalence estimates of childhood and adolescent ME/CFS to guide clinical practice and inform health care decision-making. The wide range of prevalence rates observed in the literature is concerning. This range reflects a lack of agreement about the diagnostic criteria used to identify pediatric cases and a lack of consistency in the methods used to collect data. Broad diagnostic criteria, such as the Oxford Criteria, result in inflated prevalence rates and fail to adequately distinguish true-positive cases from non-cases. Psychological and behavioral therapies continue to be tested on young patients with ME/CFS, but if children or teens are wrongly labeled as having ME/CFS and enrolled in trials of CBT or exercise therapy, findings from these studies are likely to be misleading and erroneous. Researchers need to agree on sampling strategies to identify true pediatric cases of ME/CFS and clinicians need to use a comprehensive triangulation approach to diagnose children and teenagers with ME/CFS, while carefully excluding young patients with health problems that mimic the illness.

## Author Contributions

KG conceived the paper. KG and CA contributed to the final draft. CA assisted with a systematic review of the literature discussed in this paper.

### Conflict of Interest Statement

The authors declare that the research was conducted in the absence of any commercial or financial relationships that could be construed as a potential conflict of interest.
